# Retention interval modulates motion-history biases in visual motion perception

**DOI:** 10.3758/s13414-026-03345-1

**Published:** 2026-09-23

**Authors:** Sabina Orso, Andrea Pavan

**Affiliations:** Department of Psychology, University of Bologna, Viale Berti Pichat 5, 40127 Bologna, Italy

**Keywords:** Serial dependence, Motion perception, Visual working memory, Perceptual stability

## Abstract

**Abstract:**

Serial dependence, the tendency for recent perceptual history to bias current judgments, has been proposed as a mechanism supporting perceptual stability, although its cognitive origin remains debated. We investigated serial dependence in motion direction estimation using random-dot kinematograms (RDKs). Participants reported perceived motion direction after delays of 0, 1, 3, or 6 s following stimulus offset. Trial-level linear mixed-effects modelling revealed a significant serial dependence effect and a significant interaction with delay, indicating that the magnitude of the DoG-based history effect varied across retention intervals. Contrary to accounts predicting immediate repulsion and delayed attraction, the parametric DoG analysis yielded positive peak estimates at all delays, including the nominal 0-s condition, in which no additional retention interval followed stimulus offset. However, a complementary folded-bias analysis indicated that this parametric attractive component was not uniformly expressed across angular distances but was strongest at small-to-intermediate current-minus-previous angular differences and coexisted with local repulsive deflections. These findings do not support a simple dissociation between immediate sensory repulsion and delayed mnemonic attraction. Instead, they suggest that recent motion history already influences current reports in the immediate-report condition, although this condition should not be interpreted as isolating a purely perceptual stage. Retention interval then modulated the strength and expression of this influence. Exploratory analyses further suggested that motion judgments may be influenced not only by the exact previous direction but also by the broader motion axis, whereas response-time analyses did not reveal a simple overall dependence on trial-to-trial angular similarity.

**Open practices statement:**

The raw data, analysis code, and MATLAB/Psychtoolbox experiment task code for this study are publicly available on the Open Science Framework at https://osf.io/9ea8p/. The study was not preregistered.

**Supplementary Information:**

The online version contains supplementary material available at https://doi.org/10.3758/s13414-026-03345-1.

## Introduction

Visual perception appears stable and continuous even though the visual system must constantly cope with sensory noise, eye movements, blinks, and rapid changes in retinal input. One mechanism thought to contribute to this apparent stability is serial dependence, that is, the tendency for perceptual judgments to be biased toward recently encountered stimuli. Serial dependence has been reported across several visual domains, including orientation, faces, numerosity, and motion, suggesting that it may reflect a general principle of temporal continuity in visual processing rather than a phenomenon tied to a single stimulus class (Bliss et al., 2017; Cicchini et al., 2024; Fischer & Whitney, 2014; Liberman et al., 2014; Moon et al., 2023).

A central unresolved issue concerns the cognitive locus of serial dependence. Some accounts propose that it arises at a perceptual stage, where recent sensory input is automatically integrated with current information to stabilize noisy perceptual estimates (Fischer & Whitney, 2014). In contrast, other accounts emphasize the role of post-perceptual processes, including visual short-term memory (VSTM), decision-related mechanisms, or their interaction (Bliss et al., 2017; de Azevedo Neto & Bartels, 2021; Kiyonaga et al., 2017). More broadly, recent work suggests that serial dependence may not reflect a single mechanism, but rather the combined outcome of multiple processes whose relative contribution depends on task demands, temporal structure, and stimulus history (Cicchini et al., 2024; Kiyonaga et al., 2017; Manassi et al., 2023; Pascucci et al., 2019, 2023). Recent studies propose describing serial dependence as a two-stage process: an early perceptual repulsive bias that is subsequently counteracted by a later attractive bias linked to VSTM and decision-making processes (Shan et al., 2025). The negative perceptual bias, namely the tendency for perception of the current stimulus to be repelled away from previous stimuli, seems to resemble negative adaptation effects, which serve to maximize sensitivity to change and to optimize visual processing (Fritsche et al., 2017). At present, conflicting results prevent a clear understanding of the nature of serial dependence.

Typically, variations in error magnitude that correlate with changes in the time interval between stimulus presentation and response are attributed to mnemonic processes. Accordingly, an experimental design that incorporates parametric manipulation of this interval could help understand the role of VSTM in serial dependence (Bliss et al., 2017).

Visual motion processing is a fundamental neural function, yet the literature on serial dependence in motion perception remains limited. Recent work has shown that the sequential integration of motion signals generates a pattern in which the perception of the current stimulus’s motion is biased toward the preceding one, producing an attractive effect that coexists with an early repulsive aftereffect (Moon et al., 2023). Interestingly, this study also suggests that the integration of successive motion cues may depend not only on the exact previous direction of motion but also on the broader axis of motion (Moon et al., 2023). This raises the theoretical possibility that temporal continuity in motion judgments may operate at a level broader than exact direction coding. Since the present study was not designed to provide a definitive test of motion-axis representations, we treat, this issue as an exploratory question. We therefore focus primarily on whether serial dependence in perceived motion direction varies across retention intervals.

The present study investigated whether serial dependence in perceived motion direction varies as a function of the retention interval between stimulus offset and response. Specifically, we tested whether attraction emerges primarily when visual short-term memory is engaged, whereas immediate responses are dominated by adaptation-related repulsion, or whether the temporal profile of motion-history biases is better explained by a combination of partially distinct processes.

Participants completed a motion direction estimation task using random-dot kinematograms (RDKs) and reported perceived motion direction after delays of 0, 1, 3, or 6 s following stimulus offset. If attractive serial dependence primarily depends on post-perceptual maintenance, we expected little or no attraction in the immediate-report condition and stronger attraction after nonzero retention intervals. Conversely, if recent motion history already influences current reports at immediate report, attraction should also be detectable in the 0-s condition.

In addition to the main delay-based analysis, we conducted exploratory analyses to examine whether motion-history biases were related not only to the exact previous direction but also to the broader motion axis, in line with recent evidence suggesting that motion history effects may operate beyond exact direction coding. Finally, we examined whether response times varied with trial-to-trial angular similarity and included a previous-response control analysis to assess whether response-, decision-, or motor-history components contributed to the observed bias.

## Method

### Participants

Twenty-eight participants took part in the study (20 females, eight males; mean age = 25 years, *SD* = 2.34, range = 19–30). Participants were recruited through advertisements and word of mouth within the university community. All reported normal or corrected-to-normal vision and no history of neurological or visual disorders relevant to the task. Participation was voluntary, and all participants provided written informed consent before the start of the session. The study was conducted in accordance with the Declaration of Helsinki and was approved by the Bioethics Committee of Alma Mater Studiorum – University of Bologna, Department of Psychology “Renzo Canestrari” (protocol no. 0003794).

### Apparatus

Stimuli were generated in MATLAB (MathWorks, Natick, MA, USA) using Psychtoolbox (Brainard, 1997; Kleiner et al., 2007; Pelli, 1997) and were presented on an HP Z1 G9 desktop computer connected to a 24.5-in. Dell S2522HG LCD monitor (1,920 × 1,080 resolution, 60-Hz refresh rate). Viewing distance was maintained at approximately 57 cm using a chin rest. At this distance, each pixel subtended approximately 0.03° of visual angle. The experiment was conducted in a dark and quiet room. Responses were collected using a computer mouse. The display was not gamma-corrected.

### Stimuli

The stimulus was a centrally presented random-dot kinematogram (RDK) displayed on a gray background. The RDK consisted of 200 white dots, each 6 pixels in diameter, corresponding to approximately 0.18° of visual angle, shown within a circular aperture of approximately 10° in diameter. Each RDK was presented for 0.5 s, and dot speed was approximately 6°/s. Motion coherence was fixed at 100%, so that the global motion direction was clearly visible. Dots were not assigned a limited lifetime and therefore remained visible for the full stimulus duration unless they reached the aperture boundary. When a dot moved outside the circular aperture, or within a 3-pixel margin from the aperture edge, it was repositioned at a randomly selected location within the aperture and continued moving in the assigned direction. A central fixation point (a dark disk; radius ≈ 0.15°) remained visible throughout the trial. Motion direction varied from trial to trial over the full 0–360° range. In the coordinate system used for stimulus generation and response collection, 0° corresponded to rightward motion, and angles increased clockwise.

### Procedure

The experiment consisted of a training phase followed by the main experiment.

#### Training phase

Before the main task, participants completed two training blocks of 30 trials each. In each trial, a central RDK (0.5 s, 100% coherence) was presented and followed by the response phase. During the first 24 trials of each block, target directions were selected from a set of 12 equally spaced directions (0: 30: 330°), with a minimum change of 45° between consecutive trials. In the final 6 trials, smaller directional changes were introduced (± 2°, ± 5°, ± 10°, ± 15°, ± 20°, ± 30°) relative to the previous trial’s direction. Participants reported the perceived motion direction by moving the mouse along a circular response ring centered on fixation and confirming their response with a left mouse click within 6 s. At the end of each block, performance was evaluated using two criteria: mean absolute angular error had to be ≤ 20°, and the proportion of “flip” responses (|error|> 135°) had to be ≤ 10%. Participants repeated any training block that did not meet these criteria.

#### Main experiment

The main experiment comprised 480 trials divided into eight blocks of 60 trials. Trial order was generated before the start of the experiment, and each block was run separately. At the end of each block, participants took a short break. For each trial, the design specified the current motion direction, the previous-trial motion direction, the signed angular change between consecutive targets ($\Delta \theta$), the $\Delta \theta$ bin, and the response delay (0, 1, 3, or 6 s).

The sequence of target directions was generated as a circular random walk. The first trial direction was selected randomly between 0° and 360°, and each subsequent direction was obtained by adding or subtracting a signed angular change from the previous trial, with wrap-around over the full circle. Directional transitions were sampled from three classes: small (≤ 30°; 2°, 5°, 10°, 15°, 20°, 30°), medium (30–90°; 45°, 60°, 75°, 90°), and large (> 90°; 120°, 150°, 170°). These classes were sampled with probabilities of 60%, 30%, and 10%, respectively. This was done to over-represent small angular differences where serial dependence is mostly present and to partially compensate for the small number of trials used in this experiment compared to other serial dependence studies. To avoid wrap-related artifacts, 180° transitions were excluded, and positive and negative signs of $\Delta \theta$ were approximately balanced.

The four delay conditions (0, 1, 3, and 6 s) were distributed uniformly across the experiment, with 120 trials per delay, and were randomized across the trial sequence.

**Fig. 1:**
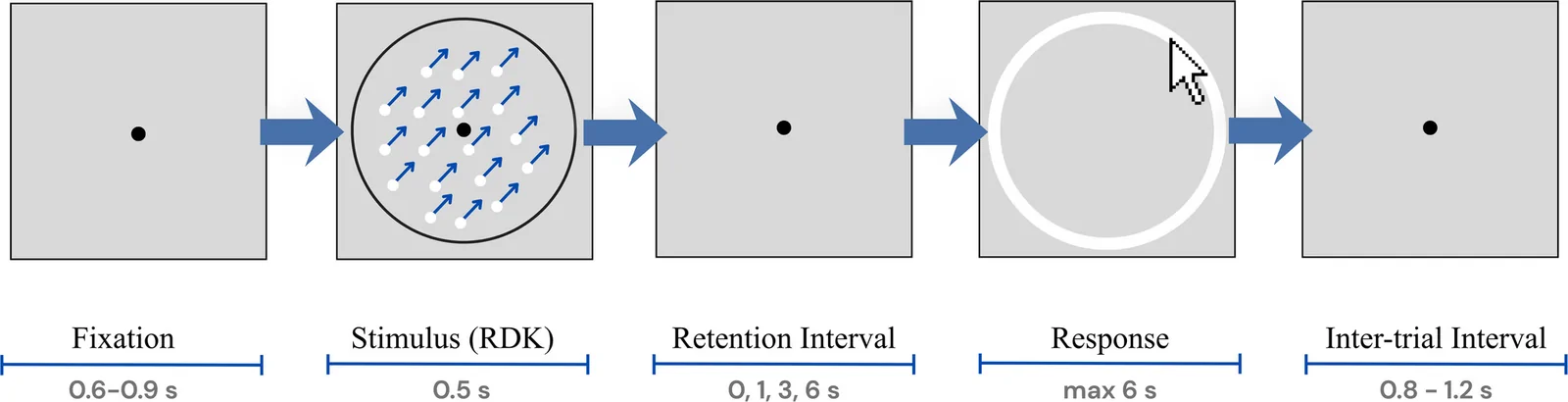
Experimental procedure. Each trial began with a central fixation point (0.6–0.9 s), followed by a random-dot kinematogram (RDK; 0.5 s). After stimulus offset, a variable retention interval was presented (0, 1, 3, or 6 s), during which only the fixation point remained visible. Participants then reported the perceived direction of motion using a circular response interface by moving the cursor along the ring and confirming their response with a left click (maximum response time: 6 s). Finally, a variable inter-trial interval (0.8–1.2 s) preceded the next trial

Each trial began with a central fixation point presented for a jittered interval of 0.6–0.9 s. The RDK was then shown centrally for 0.5 s. After stimulus offset, only fixation remained visible for a retention interval of 0, 1, 3, or 6 s depending on the trial (Fig. 1). Participants then reported the perceived motion direction using a circular response interface. The response ring had the same diameter as the stimulus aperture, and the mouse cursor was constrained to the circular region. The cursor starting angle was randomized on every trial. Participants positioned the cursor along the ring and confirmed their response with a left mouse click. The maximum response time was 6 s; if no response was given within that interval, the trial was marked as a lapse. After the response or timeout, fixation remained on screen for a jittered inter-trial interval of 0.8–1.2 s before the next trial began.

### Data analysis

The main aim of the analysis was to determine whether serial dependence in motion direction estimation varied as a function of the delay between stimulus offset and response.

The dependent variable was the angular response error on the current trial, defined as the circular difference between the reported direction and the current target direction. All angular quantities were wrapped to the interval from − 180° to 180°. Trials marked as lapses, as well as block-start trials for which no immediately preceding trial was available within the same uninterrupted block sequence, were excluded from analysis. For the main direction-based analysis, trials were also restricted to those in which the current-minus-previous angular difference fell within ± 90°. Response-error outliers in the main cleaned dataset were then removed using a pooled robust MAD-based procedure applied to signed response errors across all otherwise eligible trials. Specifically, the median and median absolute deviation (MAD) of signed response errors were computed across eligible trials, and trials whose absolute deviation from the median exceeded 3 scaled MADs were excluded. This outlier procedure defined the final cleaned dataset used in the main delay-specific inferential analyses.

Before the main delay-specific analyses, the width parameter of the derivative-of-Gaussian (DoG) function was estimated from pooled eligible trial-level response errors across all participants and delays. This preliminary sigma-estimation step was performed separately from the final main-analysis outlier exclusion, because its purpose was to obtain a stable estimate of the overall DoG tuning width rather than to define the final inferential dataset. For sigma estimation, only otherwise valid within-block trials with current-minus-previous directional differences within ± 90° were considered. A separate sigma-specific outlier filter was then applied before fitting: trials with absolute response errors greater than 120° were removed, as were trials whose response errors exceeded 4 scaled MADs within each 15° signed-$\Delta \theta$ bin. This left 11,861 trials for sigma estimation. Sigma was first estimated using a frequentist pooled fitting procedure in which, for each candidate value of σ, the corresponding DoG amplitude was solved analytically, and the residual sum of squares was minimized. This frequentist estimate was then used to initialize a Bayesian Metropolis–Hastings sampling procedure, which jointly estimated amplitude, log-sigma, and residual error variance from the pooled data. The posterior median of sigma, $\sigma = {11.26}^{\circ }$, was used as the fixed width parameter in all subsequent analyses. Pooling across participants and delays was used to obtain a stable estimate of the overall tuning width. We then fixed σ in the delay-specific mixed-effects models so that delay effects could be interpreted as changes in bias magnitude, rather than as simultaneous changes in both amplitude and curve width. We acknowledge that fixing σ is a simplifying assumption; for this reason, we complemented the DoG analysis with goodness-of-fit diagnostics and a folded-bias analysis that did not impose a DoG shape on the data.

To quantify serial dependence, we modeled response error as a function of the directional relationship between the current and previous motion stimuli using a derivative-of-Gaussian (DoG) function. The signed trial-to-trial direction change was defined as $\Delta \theta$ = current target direction − previous target direction. For the DoG analysis, the predictor was expressed as $x = -\Delta \theta$, so that x represented the previous direction relative to the current direction. The direction-based predictor was defined as:

1 $$DoG(x) = x\bullet exp\left(-\frac{{x}^{2}}{2{\sigma }^{2}}\right)$$

where x represents the previous direction relative to the current direction (i.e., $-\Delta \theta$). Consistent with standard serial-dependence analyses, only trials with $\left|x\right|\le {90}^{\circ }$ were included in the main direction-based model. The width parameter of the DoG was fixed at $\sigma = {11.26}^{\circ }$, based on the pooled estimate described above. Fixing σ allowed us to assess changes in the magnitude of serial dependence across delays while keeping the shape of the tuning function constant.

At the model level, the coefficient associated with the DoG predictor represented the amplitude of the serial-dependence effect. Under this convention, a positive amplitude indicated attraction toward the previous stimulus direction, a negative amplitude indicated repulsion, and a value of zero indicated no serial-dependence effect. For ease of interpretation, amplitudes were also converted into peak bias estimates in degrees using:

2 $$Peak = a\bullet \sigma \bullet {e}^{-1/2}$$

where a is the fitted DoG amplitude.

The primary inferential analyses were conducted at the trial level using linear mixed-effects models. Delay was treated as a categorical predictor, and the direction-based DoG term was entered both as a main effect and in interaction with delay. Random intercepts for participants and participant-specific random slopes for the DoG predictor were included in the model structure. The retained primary model was errDeg ~ delay * bDoG + (1 + bDoG | participant), where bDoG denotes the fixed-width direction-based DoG predictor. Nested model comparisons were used to test whether including the Delay × DoG interaction significantly improved model fit, thereby assessing whether the strength of serial dependence varied across retention intervals. As an additional control analysis, using retained trials with a valid immediately preceding response within the same block, we also fitted a model including an analogous DoG predictor based on previous response direction and its interaction with delay, to assess whether the observed history-dependent bias could be partly accounted for by response-, decision-, or motor-history components. The model formula was errDeg ~ delay * bStim + delay * bResp + (1 + bStim | participant), where bStim and bResp denote the previous-stimulus and previous-response DoG predictors, respectively. As a robustness check, we also fitted a more complex random-effects model allowing participant-specific random effects for the full Delay × DoG structure: errDeg ~ delay * bDoG + (1 + delay * bDoG | participant).

In addition to the main direction-based analyses, planned exploratory analyses examined whether temporal continuity in motion judgments was better characterized in relation to the exact previous motion direction, the corresponding motion axis, or both. For the exploratory motion-axis analysis, we modeled response errors over the full 0–360° circular range of $\Delta \theta$ values, where $\Delta \theta$ = current target direction − previous target direction. Two DoG predictors were included in the same model: one centered on the previous motion direction (0° relative difference) and one centered on the opposite motion direction (180° relative difference). The first component tested whether responses were biased toward the exact previous direction, whereas the second tested whether responses were also biased toward the opposite direction, in line with the axis-related analysis reported by Moon et al. (2023). This analysis was collapsed across delays and was treated as exploratory, because the stimulus sequence was optimized for the main direction-based analysis rather than for a balanced test of motion-axis effects.

As a further exploratory analysis, we examined whether response time (RT) varied as a function of the absolute angular distance between the current-trial target direction and the previous-trial target direction. This analysis was restricted to trials retained by the main preprocessing pipeline, thereby matching the cleaned dataset used for the primary direction-based serial-dependence analysis. Log-transformed RTs were then modeled using a linear mixed-effects model with fixed effects of delay, absolute angular distance, and their interaction, together with a random intercept for participant. The inferential model treated absolute angular distance as a continuous predictor; binned summaries were used only for visualization.

## Results

### Delay-dependent serial dependence in motion direction estimation

Across all participants, the dataset contained 13,440 trials. Before the main direction-based analysis, 224 block-start trials (1.67% of all trials) were excluded because the first trial of each block had no immediately preceding trial within the same uninterrupted block sequence, and 24 trials (0.18%) were excluded because they were lapses or invalid responses. A further 1,310 trials (9.75%) were outside the ± 90° range used for the main direction-based analysis, and 145 trials (1.08%) were removed as MAD-based outliers. The final dataset used for the main analysis therefore included 11,737 trials, corresponding to 87.33% of all trials. Retained trials were broadly balanced across delay conditions: 2,966 trials at 0 s, 2,842 at 1 s, 3,038 at 3 s, and 2,891 at 6 s. A detailed breakdown of exclusions by delay condition is reported in the Online Supplementary Material, Table S1.

To assess serial dependence (SD), we fitted a series of linear mixed-effects models to trial-level response errors. Adding the DoG × delay interaction to the additive delay + DoG model significantly improved fit, χ^2^(3) = 12.89, *p* =.0049, suggesting that the magnitude of SD varied across delay conditions. The corresponding interaction model included fixed effects of delay, the DoG predictor, and their interaction, together with participant-specific random intercepts and participant-specific random slopes for the DoG predictor: errDeg ~ delay * bDoG + (1 + bDoG | participant). This model had lower AIC than the additive model (82776.2 vs. 82783.1), although BIC was lower for the additive model (82849.4 vs. 82864.6). Because the Delay × DoG interaction directly tested the primary question of delay-dependent modulation, this interaction model was retained for the fixed-effect inference and delay-specific peak estimates reported below.

In the final model, there was a significant main effect of the DoG predictor, *F*(1, 28.05) = 21.44, *p* <.001, and a significant DoG × delay interaction, *F*(3, 11699.53) = 4.30, *p* =.0049, whereas the main effect of delay was not significant, *F*(3, 11693.48) = 1.61, *p* =.184. Residual diagnostics indicated that model assumptions were adequately satisfied, with residuals showing an approximately normal distribution and only minor deviations in the tails (skewness = −0.010; kurtosis = 3.072).

As a robustness check, we also fitted a more complex random-effects model that allowed participant-specific random effects for the full Delay × DoG structure, errDeg ~ delay * bDoG + (1 + delay * bDoG | participant). This model tested whether the fixed Delay × DoG interaction remained reliable when delay-specific DoG effects were also allowed to vary across participants. The model was singular, indicating that the data did not support reliable estimation of all random-effects variance components, and it was therefore not used as the primary model. Importantly, however, the fixed-effect conclusions were unchanged: the DoG main effect, *F*(1, 27.96) = 22.09, *p* <.001, and the Delay × DoG interaction, *F*(3, 90.60) = 4.30, *p* =.0070, remained significant.

**Fig. 2:**
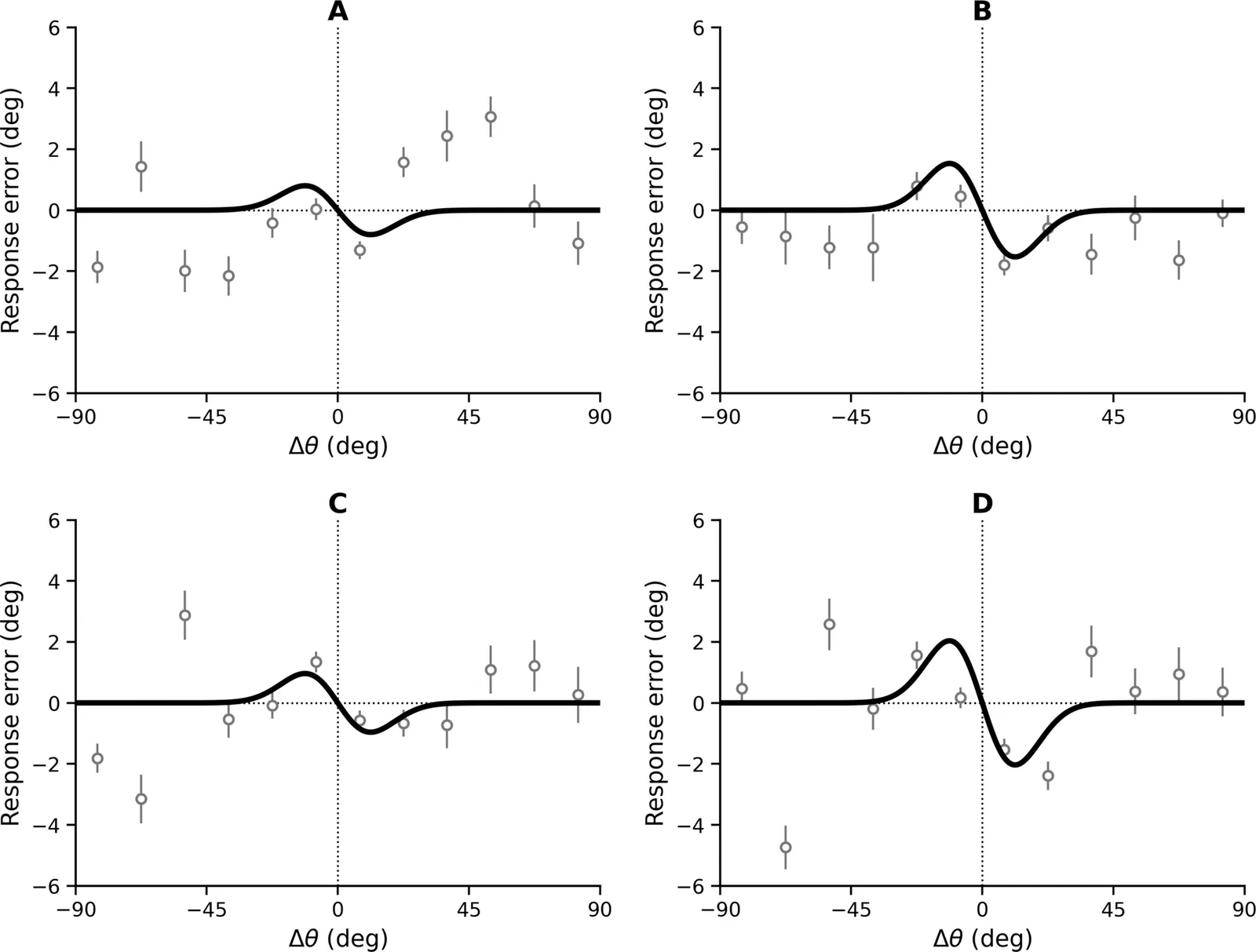
Serial dependence as a function of delay. Panels **A–D** correspond to delays of 0 s, 1 s, 3 s, and 6 s, respectively. Open circles indicate the participant-averaged mean signed response error in each 15° bin of $\Delta \theta$, where $\Delta \theta$ = current target direction − previous target direction. Error bars represent ± 1 SEM across participants. The solid black line shows the derivative-of-Gaussian (DoG) function using the fixed width parameter estimated on pooled data ($\sigma =11.26^\circ$) and the delay-specific amplitude estimated from the trial-level linear mixed-effects model. Positive response-error values indicate clockwise errors relative to the current target direction, whereas negative values indicate counterclockwise errors. Vertical and horizontal dotted lines indicate zero on the $\Delta \theta$ and response-error axes, respectively. Binning was performed over the range from − 90° to 90° using 15° bins. The fitted DoG curve summarizes the attractive component estimated by the model and does not capture all local structure visible in the binned means

**Fig. 3:**
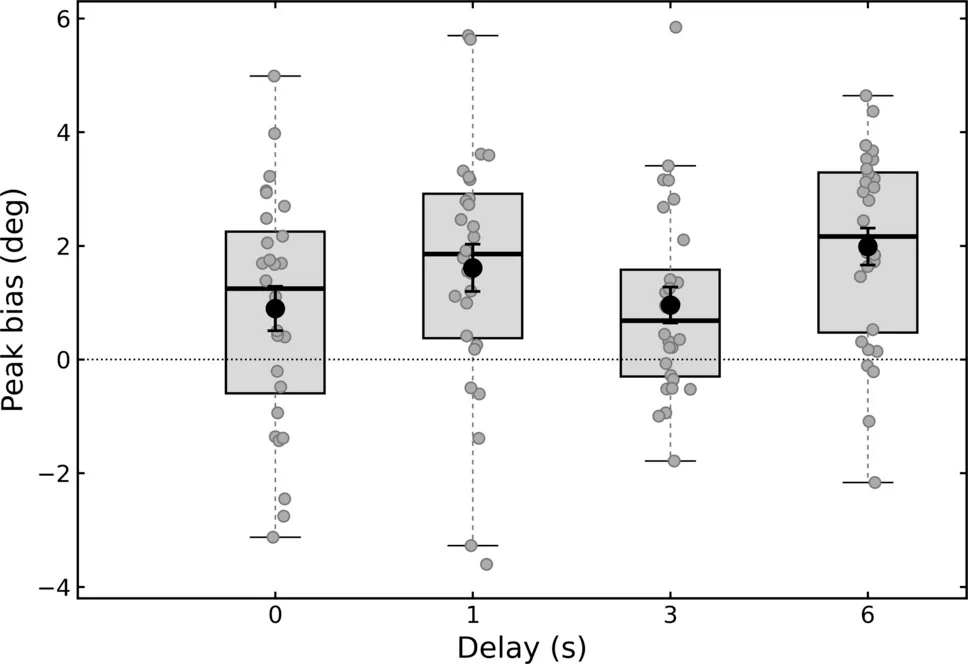
Summary of peak serial dependence across delays. Boxplots show the distribution of participant-level peak serial dependence estimates for each delay condition (0 s, 1 s, 3 s, and 6 s). The horizontal line inside each box indicates the median, boxes represent the interquartile range (IQR), and whiskers extend to the most extreme values within 1.5 × IQR. Gray points represent individual participants. Black dots indicate the group mean, and error bars represent ± 1 *SEM*. Positive values indicate attractive bias, whereas negative values indicate repulsive bias

Descriptive *SD* curves for each delay are shown in Fig. 2. The DoG-based peak estimates were positive at all delays, indicating attractive bias in the parametric model, and reached 0.80° at 0 s, 1.53° at 1 s, 0.96° at 3 s, and 2.04° at 6 s. Delay-specific tests indicated that all peak estimates were significantly greater than zero after Holm correction. Figure 3 summarizes the distribution of participant-level peak estimates across delays. Holm-corrected pairwise comparisons indicated that the DoG-based SD effect was significantly larger at 6 s than at 0 s (*p* =.0079) and 3 s (*p* =.0271), whereas the remaining comparisons were not significant.

Because the fixed-width DoG model imposes a specific parametric shape, we also conducted a complementary folded-bias analysis (Online Supplementary Material, Fig. S1 and Table S8). In this analysis, $\Delta \theta$ was defined as current target direction minus previous target direction. Positive and negative $\Delta \theta$ values were collapsed, and signed response errors were realigned so that positive folded-bias values indicated attraction toward the previous stimulus and negative values indicated repulsion away from it. Bias was then summarized as a function of the nominal absolute angular differences present in the design: 2°, 5°, 10°, 15°, 20°, 30°, 45°, 60°, 75°, and 90°. This analysis showed that attraction was not uniformly distributed across angular distances. Positive biases were most consistently observed at small-to-intermediate angular differences, particularly around 10°–20°, whereas negative deflections were also observed at some intermediate and larger distances. Thus, the folded-bias analysis indicated that recent motion history influenced current reports, while also showing that the DoG model should be interpreted as a compact parametric summary rather than as a complete description of the response-error profile.

Additional stimulus-sequence checks were conducted to address whether the non-uniform transition distribution could have encouraged distributional expectations or regression-to-the-mean-like response strategies (Online Supplementary Material, Table S9). Current target directions were broadly distributed over the full 0–360° range, with low circular concentration (R = 0.072), although 30° bin counts deviated from perfect uniformity. As intended by the design, small current-minus-previous transitions (≤ 30°) were overrepresented (60.02%), whereas transition signs were broadly balanced (49.29% positive, 50.71% negative). These checks indicate that the design did not introduce a strong global directional bias, but they also confirm that serial-dependence effects cannot be fully disentangled from expectations based on the transition structure of the sequence.

### Previous response control analysis

As an additional control analysis, we examined whether the history-dependent bias observed in the main analysis could be partly accounted for by previous-response direction. For each retained trial with a valid immediately preceding response within the same block, we computed a second DoG predictor based on the angular difference between the previous response direction and the current target direction and entered it into the model together with the original previous-stimulus DoG predictor. The previous-stimulus and previous-response predictors were moderately correlated, *r* =.680, indicating substantial but incomplete overlap between stimulus-history and response-history information. Adding the previous-response predictor and its interaction with delay substantially improved model fit relative to the stimulus-only model, χ^2^(4) = 653.64, *p* <.001. In the control model, previous-response history strongly predicted current response error, *β* = 0.694, SE = 0.027, *t* = 25.66, *p* <.001. The Delay × previous-stimulus interaction was no longer significant, *F*(3, 11,677.41) = 2.00, *p* =.111, and the Delay × previous-response interaction was also not significant, *F*(3, 11,680.96) = 1.90, *p* =.127. Although one delay-specific previous-response coefficient reached significance, the overall Delay × previous-response interaction was not significant. These results suggest that previous-response history explained additional variance in current response error, indicating that response-, decision-, and/or motor-history components contributed to the observed bias. Because previous-stimulus and previous-response histories were moderately correlated, this analysis cannot fully separate stimulus-driven and response-related contributions. Therefore, the history-dependent effects reported above should not be interpreted as purely stimulus-driven. Full results are reported in the Online Supplementary Material, Tables S2–S6.

### Exploratory analysis of motion-axis contributions to serial dependence

**Fig. 4:**
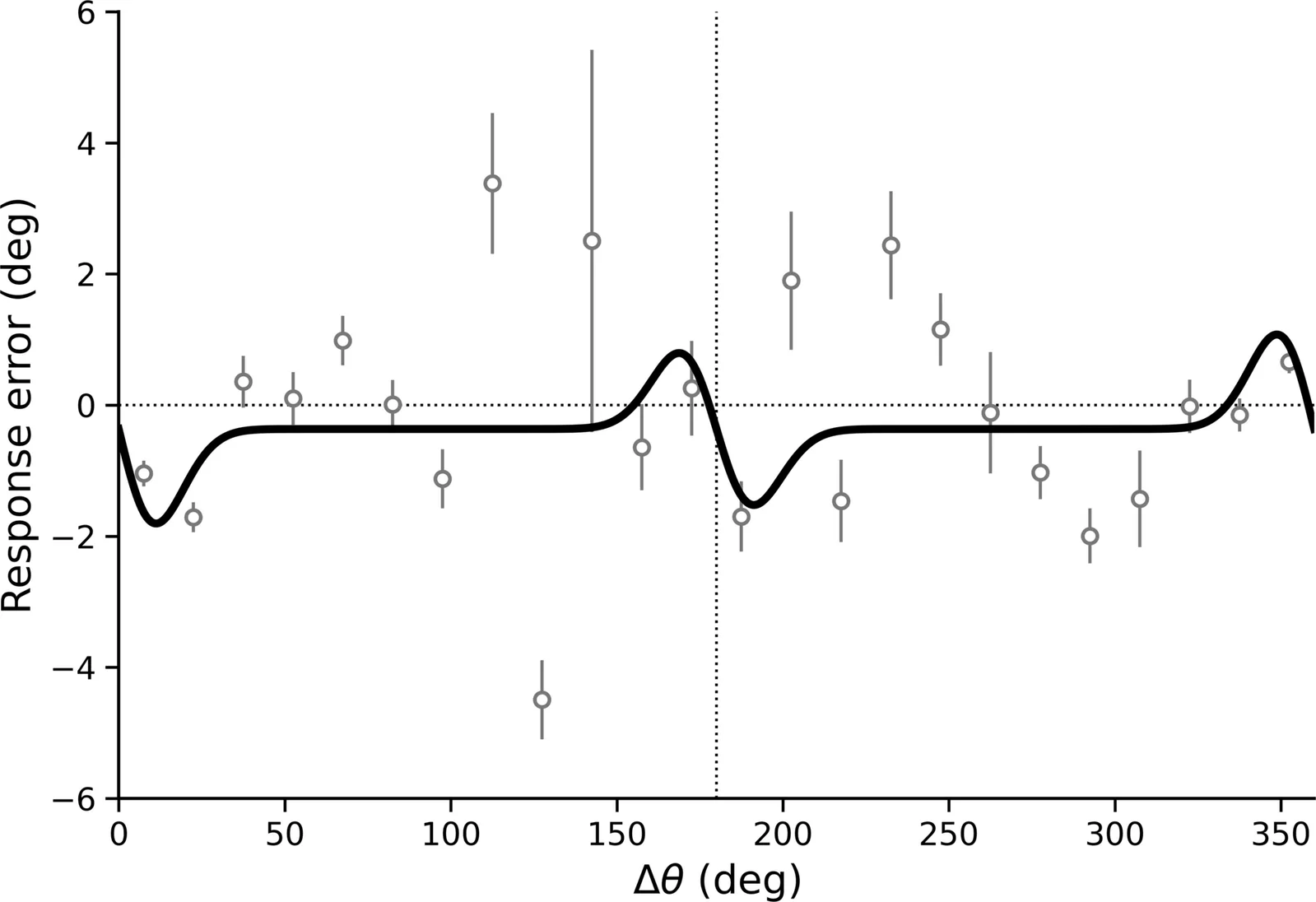
Exploratory motion-axis analysis collapsed across delays. Response errors are plotted as a function of $\Delta \theta$ over the full 0–360° circular range, where $\Delta \theta$ = current target direction – previous target direction. Thus, 0° indicates that the previous and current motion directions were the same, whereas 180° indicates opposite motion directions. Open circles indicate the mean response error across trials within each 15° angular bin, and error bars represent ± 1 *SEM* across trials. The solid black line shows the fitted two-component derivative-of-Gaussian model, with the width fixed at the value estimated from the pooled data ($\sigma =11.26^\circ$); one component was centred on 0° and the other on 180°. Because the circular scale wraps around, the component centred on 0° is visible at both the left and right edges of the plot. Positive fitted components at both 0° and 180° are consistent with the possibility that serial dependence in motion perception is influenced not only by the exact previous direction but also by the broader motion axis

To further examine whether serial dependence in motion judgments might reflect not only the exact previous motion direction but also the broader motion axis, we conducted an exploratory analysis inspired by Moon et al. (2023). Trials were collapsed across delays, and response errors were modeled over the full 0–360° range using two derivative-of-Gaussian (DoG) components: one centered on the previous direction (0° relative difference) and one centered on the opposite direction (180° relative difference; Fig. 4). Both fitted components were positive, with peak estimates of 1.44° for the 0° component and 1.16° for the 180° component. Including the 180° component significantly improved model fit relative to a direction-only model, *F*(1, 13,189) = 6.09, *p* =.0136, and reduced AIC (58,391.74 vs. 58,395.83), although BIC was slightly lower for the simpler model (58,410.80 vs. 58,414.20). Importantly, the two components did not significantly differ from each other, *t*(13,189) = 0.57, *p* =.567, with an estimated peak difference of 0.28° (95% CI [− 0.69°, 1.25°]).

Taken together, these exploratory results are consistent with the possibility that serial dependence in motion may be influenced not only by exact previous direction, but also by the broader motion axis. This interpretation should be considered hypothesis-generating rather than confirmatory. The observed 180° component is broadly compatible with recent evidence that motion direction can be represented in a bimodal format, with information related to both the true and opposite directions of motion (Chetverikov & Jehee, 2023). However, the present experiment was not designed to provide a definitive test of an axis-based serial-dependence mechanism. The design was optimized for the main direction-based serial-dependence analysis: small directional changes were overrepresented and exact 180° transitions were excluded, resulting in substantially sparser sampling of the 180° region of the circular stimulus space than of the 0° region.

### Exploratory response time analysis

**Fig. 5:**
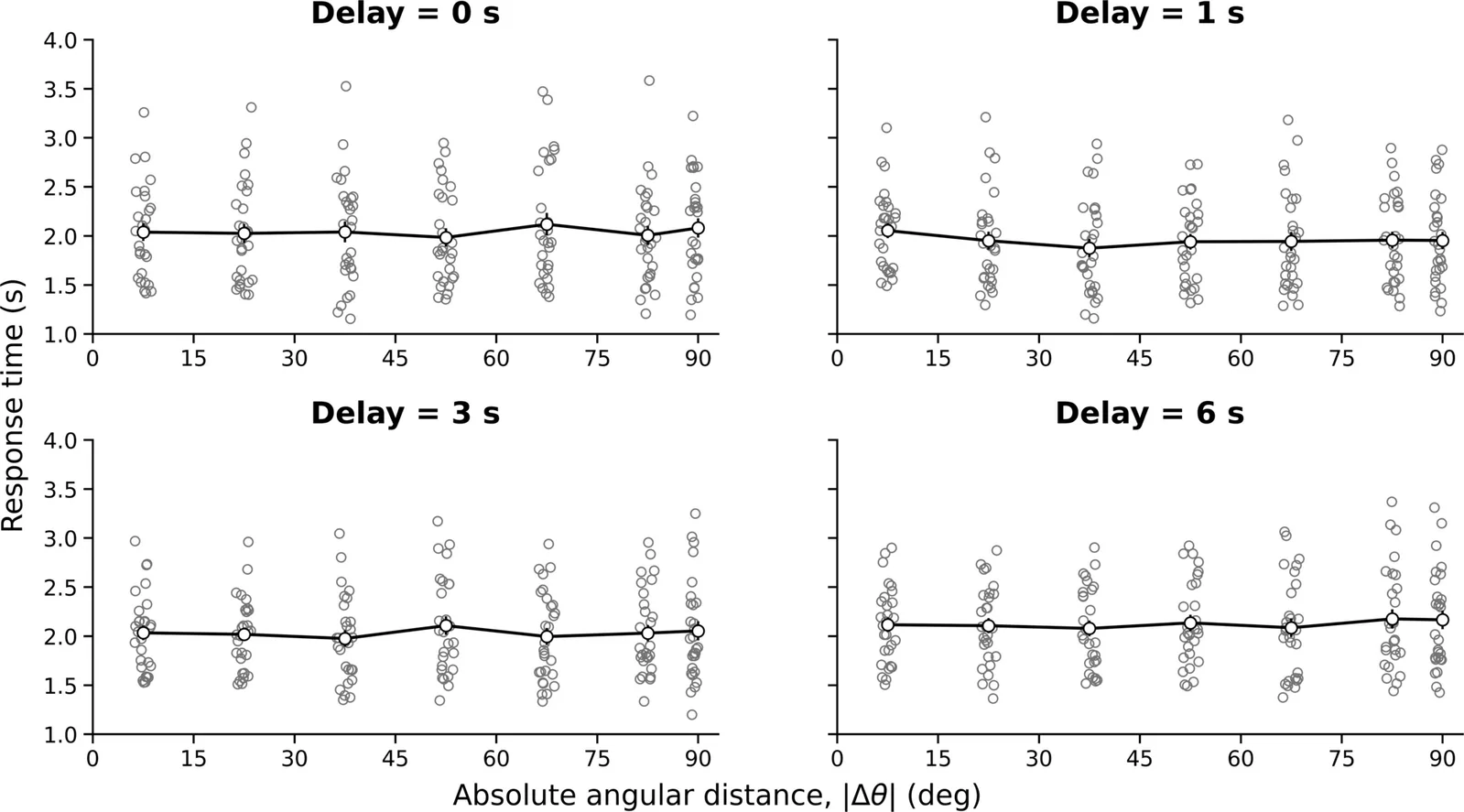
Exploratory response-time analysis. Mean response time (RT) (± *SEM*) is plotted as a function of absolute trial-to-trial angular distance, $\left|\Delta \theta \right|$, where $\Delta \theta$ is defined as current target direction minus previous target direction. For visualization, $\left|\Delta \theta \right|$ values were summarized into seven x-axis positions: six 15° intervals from 0 to < 90° and a separate 90° point. Gray open circles denote participant-level mean RTs within each delay and angular-distance bin or point, whereas black open circles and connecting lines denote the group mean ± *SEM*. Although RT differed across delay conditions and the RT–angular-distance relation varied somewhat across delays, there was no simple or consistent relation between RT and angular distance

An exploratory mixed-effects analysis of log-transformed RTs showed a significant main effect of delay, *F*(3, 11,702.11) = 9.24, *p* <.001, no main effect of absolute angular distance, *F*(1, 11,702.11) = 0.17, *p* =.681, and a significant Delay × absolute angular distance interaction, *F*(3, 11,702.19) = 3.71, *p* =.011. Thus, although RTs varied across delay conditions and the delay-specific relation between RT and angular distance was not identical across delays, there was no evidence for a simple overall dependence of RT on trial-to-trial angular similarity (Fig. 5). For visualization only, RTs were summarized across angular-distance intervals.

## Discussion

The primary aim of this study was to investigate serial dependence in visual motion processing and to explore whether different retention intervals modulate this phenomenon, thereby suggesting distinct contributions of perceptual and mnemonic processes. We asked participants to report the perceived direction of motion of successive random-dot kinematograms (RDKs) across four conditions defined by the delay between stimulus offset and response phase (0, 1, 3, and 6 s). Three main findings emerged. First, participants’ reported motion directions showed a DoG-based attractive bias toward the previous motion direction at all tested delays, including the nominal 0-s condition, in which participants responded immediately after stimulus offset, without any additional retention interval. Second, the magnitude of this DoG-based bias varied with delay, showing a non-monotonic trajectory. Third, the temporal pattern did not support a simple dissociation between immediate repulsion and delayed attraction. Taken together, these results suggest that DoG-based attractive motion-history effects are already evident at immediate report, when no additional retention interval follows stimulus offset. However, the nominal 0-s condition should not be interpreted as isolating a purely perceptual component, because responses necessarily involved post-stimulus decisional and motor stages. Rather, the findings indicate that the magnitude of serial dependence is modulated by retention delay, without supporting a simple account in which attraction emerges only after an extended retention interval.

The complementary folded-bias analysis (Online Supplementary Material, Fig. S1 and Table S8) further qualified this conclusion by showing that the attractive component summarized by the DoG model was not uniformly expressed across angular distances. Attraction was strongest at small-to-intermediate current-minus-previous angular differences, whereas local repulsive deflections emerged at some intermediate and larger distances. Therefore, the present findings are better interpreted as evidence for distance-dependent motion-history biases than as evidence for uniform attraction across the entire range of current-minus-previous direction differences.

A central implication of these findings concerns the ongoing debate about the cognitive locus of serial dependence. The observation of DoG-based attraction in the 0 s delay condition challenges strong post-perceptual accounts claiming that attraction emerges only after information is maintained in memory. At the same time, the delay-dependent modulation argues against a purely immediate sensory account. More specifically, the present pattern suggests that at least three partially distinct components may have contributed to the observed bias. First, the DoG-based attraction observed even at immediate report indicates that recent motion history influenced current judgments even when no additional retention interval was included. Second, the significant Delay × DoG interaction indicates that this influence was modulated by the retention interval, consistent with a contribution from post-stimulus maintenance, mnemonic reweighting, or decision-related processes. Third, the folded-bias analysis showed that attraction was not uniform across angular distances and coexisted with local repulsive deflections, suggesting that a single homogeneous attractive mechanism is unlikely to fully describe the data. Thus, our results do not isolate one specific mechanism, but they are more consistent with the view that serial dependence in motion reflects the combined contribution of stimulus-history, retention-related, and decision-related influences, whose relative weight may depend on task demands and temporal structure.

The previous-response control analysis further qualified this interpretation. When an analogous DoG predictor based on previous-response direction was included, previous-response history explained additional variance in current-trial errors and the Delay × previous-stimulus interaction was no longer significant. Importantly, however, previous-stimulus and previous-response histories were moderately correlated, making them difficult to disentangle within the present design. Consequently, these findings should not be interpreted as demonstrating a purely response-driven account of the effect. Rather, they suggest that stimulus-history and response-history factors likely both contributed to the observed bias, and that future studies specifically designed to decorrelate these sources of information will be needed to determine their relative contributions more precisely.

Importantly, the influence of retention interval on serial dependence was non-monotonic, indicating that its strength varied across delays rather than following a linear trend. The DoG-based attractive bias was moderate at 0 s after stimulus offset, increased at 1 s, diminished at 3 s, and reached its peak at 6 s. This temporal profile indicates that retention delay modulated the magnitude of serial dependence, but it does not support a monotonic account in which attraction steadily increases as the retention interval becomes longer. Thus, although the pattern is broadly relevant to accounts linking serial dependence to working-memory retention (Markov et al., 2024), it cautions against interpreting the delay effect as straightforward evidence for a purely retention-based mechanism.

Rather, the observed pattern may reflect the combined contribution of partially distinct processes whose relative weight changes over time. One possibility is that immediate history-dependent attraction, residual sensory interactions, and later post-perceptual influences all contribute to the final response, but with different strengths at different delays. These post-perceptual influences may include memory-related processes as well as response-, decision-, or motor-history components, as suggested by the previous-response control analysis. In this view, the retention interval does not create attraction from scratch, but changes the strength and expression of an already detectable history-dependent bias. This interpretation is broadly consistent with work linking serial dependence to post-encoding, working-memory, or memory-prioritization processes (Andriushchenko et al., 2025; Bliss et al., 2017; Fischer et al., 2025; Fritsche et al., 2017; Mei et al., 2019), and with evidence that serial dependence can involve multiple, potentially distinct history-dependent mechanisms (Czoschke et al., 2019).

The present findings also contribute to the still limited literature on serial dependence in motion perception. Motion is especially informative because it lies at the intersection of perceptual continuity, adaptation, and working memory, providing a useful domain for examining how these processes interact over time. The present study provides evidence that recent motion history can bias current judgments even at immediate report, producing a DoG-based attractive tendency whose magnitude remains sensitive to retention interval. This extends previous work showing serial dependence for motion direction across memory episodes and demonstrating that contextual information can modulate these biases (Fischer et al., 2020). More broadly, it is also consistent with evidence from other visual features showing that serial dependence can vary with stimulus uncertainty (Gallagher & Benton, 2022).

Our exploratory motion-axis analysis provides a hypothesis-generating starting point for further investigation. Consistent with Moon et al. (2023), the results suggest that motion-history effects may not be determined solely by the exact previous direction but may also reflect the broader motion axis. This possibility is compatible with evidence that motion direction can be represented in a bimodal format, with information related to both the true and opposite directions of motion (Chetverikov & Jehee, 2023). However, because the present design was optimized for the main direction-based analysis rather than for a balanced test of axis-based effects, the apparent axis-related component should be treated as preliminary exploratory evidence rather than as definitive support for a motion-axis mechanism in serial dependence.

We also examined RTs as an exploratory measure of possible decisional contributions, motivated by recent evidence that serial biases can be accompanied by systematic response-time and mouse-trajectory effects, suggesting a contribution of post-perceptual decisional processes (Janetsky et al., 2026). In the present study, RTs varied with delay, and the relation between RT and angular distance differed across delay conditions, but there was no clear main effect of angular distance. Thus, RTs did not provide strong converging evidence for a simple similarity-based decisional account in the present task. Because the experiment was optimized for direction-estimation accuracy rather than RT dynamics, this analysis should be interpreted cautiously.

Although the present design was well suited to address the main direction-based question, some constraints should be considered when interpreting the exploratory findings. The experiment was optimized primarily for the direction-based analysis and therefore does not allow a definitive comparison between direction- and motion-axis-based accounts. In addition, only four retention intervals were sampled, and the task was not designed to fully dissociate perceptual, mnemonic, response-related, and decision-related contributions. Thus, the motion-axis and RT findings should be interpreted as exploratory.

An additional constraint is that the present design cannot fully disentangle serial-dependence effects from expectations learned from the transition distribution. Because small direction changes were intentionally overrepresented, observers could in principle have used the recent distribution of transitions to guide their responses. The stimulus-sequence checks reported in the Online Supplementary Material, Table S9 indicate that target directions were broadly distributed and that positive and negative transition signs were balanced, but they do not eliminate this possible contribution. Future studies should use designs optimized to separate stimulus-history biases from distribution-learning or regression-to-the-mean effects.

More generally, the present findings should not be taken to imply that serial dependence is necessarily adaptive or beneficial. Recent large-scale evidence suggests that serial dependence does not necessarily improve perceptual decision-making and may, in some contexts, even deteriorate performance (Ozkirli et al., 2026). Rather, the present results show that recent motion history systematically biases current judgments; whether this bias improves or worsens performance likely depends on the structure of the task and the demands placed on the observer.

In conclusion, the present study shows that DoG-based attractive motion-history effects in motion direction estimation are evident even at immediate report, when no additional retention interval follows stimulus offset. The magnitude of this bias varied across retention intervals, but the temporal profile was non-monotonic, with the largest estimate observed at 6 s rather than a steady increase across delays. Thus, the findings do not support a simple account in which immediate responses are dominated by repulsion and delayed responses uniquely reveal attraction, nor do they support a monotonic account in which serial dependence steadily increases with longer retention intervals. Instead, they suggest that recent motion history already influences current judgments at immediate report, while retention delay modulates the strength of that influence over time. Moreover, the previous-response control analysis indicates that this history-dependent bias cannot be interpreted as purely stimulus-driven. The exploratory motion-axis and RT analyses further suggest that temporal continuity in motion judgments may involve mechanisms beyond a simple direction-based account, although both extensions require confirmation in designs optimized specifically for those purposes.

## Supplementary Information

Below is the link to the electronic supplementary material.

Supplementary file1 (DOCX 1628 kb)

## Data Availability

Data associated with this study are available on the Open Science Framework: https://osf.io/9ea8p/.

## Code availability

The MATLAB/Psychtoolbox experiment scripts and analysis code are available on the Open Science Framework: https://osf.io/9ea8p/.
