# Supplementary material for: Retention interval modulates motion-history biases in visual motion perception
Source: Atten Percept Psychophys. 2026 Sep 23;88(7):191. doi: 10.3758/s13414-026-03345-1 (PMC13601103; doi:10.3758/s13414-026-03345-1)
Supplement: Supplementary file 1 [file 13414_2026_3345_MOESM1_ESM.docx]

**Supplementary Material**

*Retention interval modulates motion-history biases in visual motion perception*

**S1. Trial-exclusion summary for the main direction-based analysis**

The preprocessing pipeline excluded the first trial of every block, for which no uninterrupted previous-trial direction was available after the between-block break, as well as trials marked as lapses or invalid responses, trials with an absolute current–previous angular difference greater than 90° (|Δθ| > 90°), and MAD-based outliers. Table S1 reports the number and percentage of excluded and retained trials overall and separately for each delay condition.

**Table S1.** Trial-exclusion summary overall and by delay condition. Percentages are computed relative to the total number of trials in each row.

| **Delay** | **Total trials** | **No valid previous trial** | **Lapse/invalid response** | **Outside ±90°** | **MAD outliers** | **Final retained** | **Retained (%)** |
| --- | --- | --- | --- | --- | --- | --- | --- |
| 0 s | 3,360 | 54 (1.61%) | 5 (0.15%) | 311 (9.26%) | 24 (0.71%) | 2,966 | 88.27 |
| 1 s | 3,360 | 75 (2.23%) | 6 (0.18%) | 407 (12.11%) | 30 (0.89%) | 2,842 | 84.58 |
| 3 s | 3,360 | 34 (1.01%) | 6 (0.18%) | 243 (7.23%) | 39 (1.16%) | 3,038 | 90.42 |
| 6 s | 3,360 | 61 (1.82%) | 7 (0.21%) | 349 (10.39%) | 52 (1.55%) | 2,891 | 86.04 |
| Overall | 13,440 | 224 (1.67%) | 24 (0.18%) | 1,310 (9.75%) | 145 (1.08%) | 11,737 | 87.33 |

**Note.** The “outside ±90°” count refers to otherwise-valid trials with |Δθ| > 90° in the main direction-based analysis. MAD outliers were identified after applying the |Δθ| ≤ 90° restriction. The first trial of each block was classified as having no valid previous trial because participants took a short break between blocks. Percentages in the final column refer to the proportion of total trials retained within each delay condition. Exclusion counts follow the sequential preprocessing steps used for the main analysis.

**S2. Previous response control analysis**

**Rationale**

The main direction-based analysis tested whether current response errors were systematically related to the previous stimulus direction. However, serial-dependence effects can also reflect previous response, decision, or motor history. To address this issue, we conducted an additional control analysis in which the original previous-stimulus DoG predictor was entered together with an analogous predictor based on the previous response direction.

**Trial inclusion and predictor construction**

The analysis was conducted on trials retained by the main direction-based preprocessing pipeline (keepMask = 1). We additionally required a valid response on the immediately preceding trial within the same block, because the control analysis required the previous response direction. Cross-block response lags were therefore never used. This resulted in 11,716 trials from 28 participants.

For each trial, we computed two signed angular distances relative to the current target direction. The previous-stimulus distance was defined as the circular difference between the previous stimulus direction and the current target direction (equivalent to −Δθ, where Δθ is current minus previous target direction). The previous-response distance was defined as the circular difference between the previous response direction and the current target direction. Both quantities were wrapped to the interval from −180° to 180°. For consistency with the main direction-based analysis, trials were restricted to previous-stimulus distances within ±90°.

Both angular distances were transformed into derivative-of-Gaussian predictors using the same fixed width parameter as in the final main analysis, σ = 11.262755734066°. The previous-stimulus predictor is referred to as bStim, and the previous-response predictor as bResp. The two predictors were moderately correlated, *r* = .680, indicating that previous-stimulus and previous-response histories were related but not identical.

**Models**

We first fitted a stimulus-only model matching the structure of the main direction-based analysis:

errDeg ~ delay * bStim + (1 + bStim | participant)

We then fitted a control model including both previous-stimulus and previous-response predictors:

errDeg ~ delay * bStim + delay * bResp + (1 + bStim | participant)

Models were fitted using maximum likelihood. Delay was treated as a categorical factor with four levels: 0, 1, 3, and 6 s. The dependent variable was current-trial signed response error in degrees. Satterthwaite degrees of freedom were used for fixed-effect tests.

As an additional robustness check, we fitted a richer random-slope model including participant-specific slopes for both bStim and bResp:

errDeg ~ delay * bStim + delay * bResp + (1 + bStim + bResp | participant)

This richer model converged and was not singular.

**Results**

Adding previous-response direction substantially improved model fit relative to the stimulus-only model, χ²(4) = 653.64, *p* < .001. In the control model, previous-response history strongly predicted current response error. The previous-stimulus predictor also remained significant, but its coefficient became negative after inclusion of the previous-response predictor. Because the two predictors were moderately correlated, this negative coefficient should be interpreted cautiously and may reflect shared variance or suppression rather than a genuine repulsive stimulus-history effect.

Neither the Delay × previous-stimulus interaction nor the Delay × previous-response interaction was significant in the control model. Thus, previous-response, decision, and/or motor history explained substantial additional variance in current response error and contributed to the history-dependent bias observed in the main analysis. Because previous-stimulus and previous-response histories were moderately correlated, the present analysis cannot fully separate stimulus-driven and response-related contributions. Accordingly, the main effect should not be interpreted as purely stimulus-driven.

The richer random-slope model produced the same qualitative pattern. It improved model fit relative to the simpler control model, χ²(3) = 33.04, *p* < .001, and was not singular. In this model, both bResp and bStim were significant, whereas neither interaction with delay reached significance.

**Table S2.** Model fit statistics for the previous-response control analysis.

| **Model** | **N** | **AIC** | **BIC** | **logLik** | **Deviance** |
| --- | --- | --- | --- | --- | --- |
| Stimulus-only | 11,716 | 82,616.76 | 82,705.19 | −41,296.38 | 82,592.76 |
| Stimulus + previous response | 11,716 | 81,971.13 | 82,089.03 | −40,969.56 | 81,939.13 |
| Stimulus + previous response, richer random slopes | 11,716 | 81,944.09 | 82,084.09 | −40,953.04 | 81,906.09 |

**Note.** All models were fitted using maximum likelihood. The richer random-slope model included participant-specific slopes for both bStim and bResp.

**Table S3.** Likelihood-ratio model comparisons.

| **Comparison** | **χ²** | **df** | **p** |
| --- | --- | --- | --- |
| Stimulus + previous response vs. stimulus-only | 653.64 | 4 | < .001 |
| Richer random-slope model vs. simpler control model | 33.04 | 3 | < .001 |

**Note.** The first comparison tests whether adding previous-response direction improved model fit relative to the stimulus-only model. The second comparison tests whether adding a participant-specific random slope for bResp improved the control model.

**Table S4.** Type III ANOVA tables for the previous-response control models.

| **Model** | **Term** | **F** | **df1** | **df2** | **p** |
| --- | --- | --- | --- | --- | --- |
| Stimulus-only | delay | 1.67 | 3 | 11672.49 | .171 |
| Stimulus-only | bStim | 21.09 | 1 | 28.04 | < .001 |
| Stimulus-only | delay × bStim | 4.02 | 3 | 11678.57 | .007 |
| Control | delay | 0.75 | 3 | 11671.52 | .521 |
| Control | bStim | 34.38 | 1 | 38.83 | < .001 |
| Control | bResp | 658.30 | 1 | 11690.62 | < .001 |
| Control | delay × bStim | 2.00 | 3 | 11677.41 | .111 |
| Control | delay × bResp | 1.90 | 3 | 11680.96 | .127 |

**Note.** Satterthwaite degrees of freedom are reported. Table S4 contains the stimulus-only and primary stimulus-plus-response models. bStim = derivative-of-Gaussian predictor based on previous stimulus direction; bResp = derivative-of-Gaussian predictor based on previous response direction.

**Table S5.** Fixed-effect coefficients for the primary previous-response control model.

| **Term** | **Estimate** | **SE** | **df** | **t** | **p** |
| --- | --- | --- | --- | --- | --- |
| Intercept | −0.313 | 0.191 | 28.08 | −1.64 | .112 |
| delay 0 s | −0.070 | 0.128 | 11681.78 | −0.55 | .584 |
| delay 1 s | −0.124 | 0.129 | 11661.72 | −0.96 | .338 |
| delay 3 s | 0.166 | 0.126 | 11672.55 | 1.32 | .188 |
| bStim | −0.277 | 0.047 | 38.83 | −5.86 | < .001 |
| bResp | 0.694 | 0.027 | 11690.62 | 25.66 | < .001 |
| 0 s × bStim | −0.018 | 0.047 | 11680.52 | −0.39 | .697 |
| 1 s × bStim | 0.009 | 0.048 | 11672.91 | 0.20 | .843 |
| 3 s × bStim | −0.085 | 0.045 | 11673.66 | −1.88 | .060 |
| 0 s × bResp | −0.100 | 0.046 | 11679.27 | −2.16 | .031 |
| 1 s × bResp | 0.018 | 0.049 | 11680.57 | 0.38 | .707 |
| 3 s × bResp | 0.072 | 0.045 | 11682.41 | 1.62 | .105 |

**Note.** Delay was sum-coded. The displayed delay coefficients correspond to the 0-, 1-, and 3-s levels; the 6-s coefficient is constrained to the negative sum of those three coefficients. The model formula was errDeg ~ delay * bStim + delay * bResp + (1 + bStim | participant).

**Table S6.** Type III omnibus tests for the richer random-slope control model.

| **Term** | **F** | **df1** | **df2** | **p** | **Interpretation** |
| --- | --- | --- | --- | --- | --- |
| delay | 0.70 | 3 | 11663.39 | .550 | not significant |
| bStim | 48.83 | 1 | 27.63 | < .001 | significant |
| bResp | 228.30 | 1 | 28.87 | < .001 | significant |
| delay × bStim | 2.10 | 3 | 11629.71 | .098 | not significant |
| delay × bResp | 2.01 | 3 | 11626.28 | .111 | not significant |

**Note.** Satterthwaite degrees of freedom are reported. The model formula was errDeg ~ delay * bStim + delay * bResp + (1 + bStim + bResp | participant). This sensitivity model converged and was not singular.

**S3. Goodness-of-fit of the parametric DoG analysis**

**Rationale and method**

To address the descriptive adequacy of the parametric derivative-of-Gaussian (DoG) model, we reran the main DoG analysis and computed goodness-of-fit indices for the fitted curves. The analysis used the 11,737 trials retained by the final block-reset preprocessing pipeline (keepMask = 1). The signed current–previous direction difference was Δθ = current target direction minus previous target direction; the DoG predictor used −Δθ so that positive slopes indicated attraction toward the previous target. The fixed DoG width was σ = 11.262755734066°, matching the final main analysis.

Goodness-of-fit was evaluated on 15° binned observed means separately for each delay condition. Predictions were obtained from the final mixed-effects DoG model with delay-specific slopes, after removing delay-specific intercepts so that the comparison isolated the DoG-shaped component. Weighted indices used the number of trials in each bin as weights.

**Results**

The reanalysis reproduced the inferential pattern. The final random-slope model showed a significant omnibus DoG effect, *F*(1, 28.05) = 21.44, *p* < .001, and a significant Delay × DoG interaction, *F*(3, 11699.53) = 4.30, *p* = .0049. A likelihood-ratio comparison confirmed that including the Delay × DoG interaction improved fit relative to the additive model, χ²(3) = 12.89, *p* = .0049. Peak-bias estimates were 0.80° at 0 s, 1.53° at 1 s, 0.96° at 3 s, and 2.04° at 6 s.

However, the goodness-of-fit diagnostics indicated that the fixed-shape DoG model provided only a partial descriptive account of the binned response-error curves (Table S7), especially in the 0-s condition, where the weighted R² was low. The model therefore provides a compact parametric summary of the attractive component but does not capture all local structure in the binned data.

**Table S7.** Goodness-of-fit diagnostics for the parametric DoG analysis.

| **Delay** | **N trials** | **Peak bias (°)** | **Weighted RMSE (°)** | **Weighted MAE (°)** | **Weighted R²** | **Pearson r** |
| --- | --- | --- | --- | --- | --- | --- |
| Overall | 11,737 | NA | 1.22 | 0.86 | 0.214 | 0.276 |
| 0 s | 2,966 | 0.80 | 1.33 | 1.02 | 0.035 | 0.047 |
| 1 s | 2,842 | 1.53 | 0.68 | 0.48 | 0.535 | 0.501 |
| 3 s | 3,038 | 0.96 | 1.07 | 0.70 | 0.175 | 0.245 |
| 6 s | 2,891 | 2.04 | 1.59 | 1.26 | 0.205 | 0.317 |

**Note.** Goodness-of-fit was computed on 15° binned observed means. Weighted indices were computed using bin-wise trial counts as weights. Peak-bias values are delay-specific estimates from the final DoG mixed-effects model. The overall row summarizes goodness of fit across all delay-bin points and therefore has no single peak-bias estimate. Positive peak-bias values indicate attraction toward the previous stimulus direction.

**S4. Complementary folded-bias analysis**

**Rationale and method**
As a complementary check on the descriptive adequacy of the parametric DoG analysis, we conducted a folded-bias analysis using the same 11,737 trials retained by the final block-reset preprocessing pipeline. The purpose of this analysis was to examine the direction of bias directly at each nominal absolute angular transition without imposing a DoG shape on the data.
For each trial, the signed angular difference between the current and previous target direction was coded as:

Δθ = current target direction − previous target direction.

The signed response error was coded as:

response error = reported direction − current target direction.

Because Δθ was coded as current minus previous direction, attraction toward the previous stimulus has opposite signs depending on whether the current direction was clockwise or counterclockwise relative to the previous one. For example, if the current direction was clockwise from the previous direction (positive Δθ), attraction toward the previous direction corresponds to a negative response error. Conversely, if the current direction was counterclockwise from the previous direction (negative Δθ), attraction corresponds to a positive response error.

To express both cases on a common scale, response errors were realigned using:

folded bias = −sign(Δθ) × response error

With this convention, positive folded-bias values indicate attraction toward the previous stimulus, negative values indicate repulsion away from the previous stimulus, and values close to zero indicate no systematic bias. Positive and negative angular differences were then collapsed, and folded bias was summarized at the nominal absolute angular transitions present in the design: 2°, 5°, 10°, 15°, 20°, 30°, 45°, 60°, 75°, and 90°.

## For statistical assessment, we first computed each participant’s mean folded bias separately for each delay and nominal angular transition. We then conducted one-sample t-tests against zero for each delay × transition condition. These tests assessed whether the observed group-level mean bias in each condition deviated significantly from zero (indicating no bias). *p* values were corrected using the Holm method across the 40 tests corresponding to the 4 delays × 10 angular transitions.

**Results**

The folded-bias analysis showed that the history-dependent bias was not uniform across angular distances (Figure S1; Table S8). Positive biases were most consistently observed at small-to-intermediate angular differences, especially around 10°–20°. Local negative deflections were also present at some angular distances, including larger separations. Thus, this complementary analysis supports the interpretation that the DoG model provides a compact parametric summary of the history-dependent bias, but not a complete description of the local structure in the response-error profile.

**Figure S1.** Folded bias as a function of nominal absolute angular transition and response delay. Positive values indicate attraction toward the previous stimulus, whereas negative values indicate repulsion away from it. Points represent the group mean folded bias at each nominal absolute angular transition, where Δθ denotes the current target direction minus the previous target direction. Error bars indicate ±1 SEM across participants.

**Table S8**. Folded bias at nominal angular transitions by delay. Positive values indicate attraction toward the previous stimulus; negative values indicate repulsion away from it. For each delay × transition condition, the mean bias and SEM were computed across the 28 participant-level mean folded-bias values. The *t* values correspond to one-sample t-tests against zero, testing whether folded bias differed from no bias in that condition. *p*Holm values are Holm-corrected p values across the 40 delay × transition tests.

| **Delay** | **\|Δθ\|** | **Mean bias (°)** | **SEM** | **t** | **pHolm** |
| --- | --- | --- | --- | --- | --- |
| 0 s | 2° | −1.44 | 0.40 | −3.62 | .034 |
| 0 s | 5° | 1.62 | 0.44 | 3.70 | .031 |
| 0 s | 10° | 1.84 | 0.39 | 4.73 | .002 |
| 0 s | 15° | 1.11 | 0.54 | 2.04 | .914 |
| 0 s | 20° | −1.83 | 0.62 | −2.96 | .145 |
| 0 s | 30° | −1.52 | 0.41 | −3.68 | .031 |
| 0 s | 45° | −2.68 | 0.83 | −3.24 | .081 |
| 0 s | 60° | −0.58 | 0.46 | −1.27 | 1.000 |
| 0 s | 75° | −1.11 | 0.48 | −2.32 | .589 |
| 0 s | 90° | −0.29 | 0.63 | −0.46 | 1.000 |
| 1 s | 2° | 0.67 | 0.46 | 1.46 | 1.000 |
| 1 s | 5° | 0.98 | 0.32 | 3.10 | .108 |
| 1 s | 10° | 0.87 | 0.49 | 1.77 | 1.000 |
| 1 s | 15° | 2.59 | 0.61 | 4.26 | .008 |
| 1 s | 20° | 1.09 | 0.39 | 2.82 | .197 |
| 1 s | 30° | −2.76 | 0.82 | −3.37 | .063 |
| 1 s | 45° | 0.30 | 0.47 | 0.64 | 1.000 |
| 1 s | 60° | −0.24 | 0.69 | −0.35 | 1.000 |
| 1 s | 75° | 1.25 | 0.59 | 2.11 | .845 |
| 1 s | 90° | −0.18 | 0.36 | −0.50 | 1.000 |
| 3 s | 2° | 1.26 | 0.37 | 3.37 | .063 |
| 3 s | 5° | −0.27 | 0.40 | −0.68 | 1.000 |
| 3 s | 10° | 2.35 | 0.41 | 5.69 | < .001 |
| 3 s | 15° | −0.72 | 0.35 | −2.03 | .914 |
| 3 s | 20° | 1.82 | 0.49 | 3.69 | .031 |
| 3 s | 30° | −0.34 | 0.36 | −0.95 | 1.000 |
| 3 s | 45° | 1.71 | 0.78 | 2.20 | .738 |
| 3 s | 60° | −1.86 | 0.46 | −4.04 | .014 |
| 3 s | 75° | −1.75 | 0.56 | −3.13 | .104 |
| 3 s | 90° | −1.02 | 0.59 | −1.74 | 1.000 |
| 6 s | 2° | 0.01 | 0.47 | 0.03 | 1.000 |
| 6 s | 5° | −0.94 | 0.52 | −1.80 | 1.000 |
| 6 s | 10° | 1.57 | 0.33 | 4.70 | .002 |
| 6 s | 15° | 2.44 | 0.47 | 5.14 | < .001 |
| 6 s | 20° | 2.19 | 0.50 | 4.35 | .006 |
| 6 s | 30° | 0.46 | 0.50 | 0.92 | 1.000 |
| 6 s | 45° | 0.61 | 0.77 | 0.79 | 1.000 |
| 6 s | 60° | −2.78 | 0.70 | −3.99 | .015 |
| 6 s | 75° | −0.76 | 0.69 | −1.10 | 1.000 |
| 6 s | 90° | 0.08 | 0.45 | 0.18 | 1.000 |

**Note**. Bias values are participant-level group means computed after folding signed angular differences and realigning errors so that positive values indicate attraction toward the previous stimulus. Each condition includes 28 participants. Holm correction was applied across all 40 tests: 4 delays × 10 angular transitions.

**S5. Stimulus-distribution checks**

**Rationale and method**

To address whether the non-uniform stimulus sequence could have encouraged distributional expectations or regression-to-the-mean-like response strategies, we quantified the distribution of current target directions and valid within-block trial-to-trial directional transitions. Current target directions were summarized in 30° bins over the full 0–360° range and by circular summary statistics. Transition analyses excluded the first trial of every block and summarized absolute transition magnitude and transition sign.

**Results**

Current target directions were distributed around the full circle, although the 30° bin counts deviated from perfect uniformity (Table S9). Importantly, circular concentration was low (*R* = 0.0716), indicating no strong global clustering around a single direction. As intended by the experimental design, the within-block transition distribution was non-uniform, with small transitions (≤30°) overrepresented. However, positive and negative transition signs were approximately balanced. These checks indicate that the main sequence non-uniformity concerned transition magnitude rather than a directional sign bias. They do not rule out the possibility that observers learned aspects of the transition distribution; accordingly, this possibility is treated as a limitation in the manuscript.

**Table S9.** Stimulus-distribution and transition-distribution checks.

| **Check** | **Result** |
| --- | --- |
| Current target direction distribution | χ²(11) = 270.03, *p* < .001; 30° bin percentages ranged from 6.13% to 10.39% |
| Circular concentration of target directions | Mean resultant length R = 0.0716; mean direction = 181.82° |
| Small transitions (≤30°) | 7,932 trials, 60.02% |
| Medium transitions (>30° to 90°) | 3,971 trials, 30.05% |
| Large transitions (>90°) | 1,313 trials, 9.93% |
| Positive transition signs | 6,514 trials, 49.29% |
| Negative transition signs | 6,702 trials, 50.71% |

**Note.** Current target direction uniformity was tested using 12 bins of 30° each. Circular concentration was summarized using the mean resultant length R, where values close to zero indicate weak or absent clustering around a single direction. Transition classes and signs were calculated only for valid within-block transitions; the first trial of each block was excluded. Transition classes refer to |Δθ|, where Δθ is current target direction minus previous target direction.
